# Inhibition of Endoplasmic Reticulum Stress Improves Chronic Ischemic Hippocampal Damage Associated with Suppression of IRE1α/TRAF2/ASK1/JNK-Dependent Apoptosis

**DOI:** 10.1007/s10753-024-01989-5

**Published:** 2024-02-24

**Authors:** Kai Kang, Shu-Hui Chen, Da-Peng Wang, Feng Chen

**Affiliations:** 1 School of Public Health, Fudan University, Shanghai, 200032 China; 2 Department of Research and Surveillance Evaluation, Shanghai Municipal Center for Health Promotion, Shanghai, 200040 China; 3 Department of Radiation Oncology, Jiangxi Key Laboratory of Translational Cancer Research, Jiangxi Cancer Hospital, The Second Affiliated Hospital of Nanchang Medical College, Jiangxi Cancer Institute, Nanchang, 330029 Jiangxi China; 4 Department of Neurosurgery, Center of Pituitary Tumor, Ruijin Hospital, Shanghai Jiao Tong University School of Medicine, Shanghai, 200025 China; 5 Department of Neurosurgery, Tong Ji Hospital, Tong Ji University School of Medicine, Shanghai, 200065 China

**Keywords:** chronic cerebral hypoperfusion, endoplasmic reticulum stress, ischemic neuronal injury, ASK1/JNK signaling, apoptosis

## Abstract

Chronic cerebral ischemia is a complex form of stress, of which the most common hemodynamic characteristic is chronic cerebral hypoperfusion (CCH). Lasting endoplasmic reticulum (ER) stress can drive neurological disorders. Targeting ER stress shows potential neuroprotective effects against stroke. However, the role of ER stress in CCH pathological processes and the effects of targeting ER stress on brain ischemia are unclear. Here, a CCH rat model was established by bilateral common carotid artery occlusion. Rats were treated with 4-PBA, URB597, or both for 4 weeks. Neuronal morphological damage was detected using hematoxylin–eosin staining. The expression levels of the ER stress–ASK1 cascade-related proteins GRP78, IRE1α, TRAF2, CHOP, Caspase-12, ASK1, p-ASK1, JNK, and p-JNK were assessed by Western blot. The mRNA levels of TNF-α, IL-1β, and iNOS were assessed by RT-PCR. For oxygen–glucose deprivation experiments, mouse hippocampal HT22 neurons were used. Apoptosis of the hippocampus and HT22 cells was detected by TUNEL staining and Annexin V-FITC analysis, respectively. CCH evoked ER stress with increased expression of GRP78, IRE1α, TRAF2, CHOP, and Caspase-12. Co-immunoprecipitation experiments confirmed the interaction between TRAF2 and ASK1. ASK1/JNK signaling, inflammatory cytokines, and neuronal apoptosis were enhanced, accompanied by persistent ER stress; these were reversed by 4-PBA and URB597. Furthermore, the ASK1 inhibitor GS4997 and 4-PBA displayed synergistic anti-apoptotic effects in cells with oxygen–glucose deprivation. In summary, ER stress-induced apoptosis in CCH is associated with the IRE1α/TRAF2/ASK1/JNK signaling pathway. Targeting the ER stress–ASK1 cascade could be a novel therapeutic approach for ischemic cerebrovascular diseases.

## Introduction

Ischemic cerebrovascular disease is mainly caused by system hypoperfusion, cerebral vascular stenosis, thrombosis or embolism, and cerebral small vessel disease [1]. Chronic cerebral hypoperfusion (CCH) is the most common hemodynamic characteristic of these diseases. Early-phase CCH is generally concealed, while a sustained decrease in cerebral blood flow (CBF) can result in headaches, dizziness, cognitive decline, and irreversible neurological damage [2, 3]. Statistics show that 70% of patients with CCH are elderly people, and CCH is the most common reason for memory dysfunction [2]. Although the chronic loss of CBF to the hippocampus, which is critically involved in learning and memory, must feature as the main driver of vascular dementia or Alzheimer's disease (AD), the underlying mechanisms and interactions with related disease processes remain to be fully elucidated [4].

The endoplasmic reticulum (ER), a dynamic organelle, is involved in protein folding, trafficking, and degradation in physiological conditions [5]. Disruption of ER structure and function usually contributes to abnormal protein aggregation, triggering the unfolded protein response (UPR) in many neurological disorders, such as AD, traumatic brain injury, and cerebral ischemia/reperfusion injury [6, 7]. The UPR is a cellular quality control mechanism to improve protein folding. However, a continuous UPR can activate ER stress and apoptosis [8]. In a middle cerebral artery occlusion stroke animal model, ER stress-induced apoptosis leads to enlarged infarct size, which ultimately aggravates neurological deficits [9]. Suppression of ER stress can alleviate early brain injury in acute subarachnoid hemorrhage rats [10]. However, the role of ER stress in the pathological process of chronic cerebral ischemia is rarely reported, and it remains unclear if it may serve as a therapeutic target for preventing or treating ischemia-induced brain damage.

To clarify these issues, in the present study, the role of ER stress during CCH and the effects of regulating ER stress on brain ischemia were investigated.

## Methods

### Animals

Thirty-five male Sprague–Dawley rats, aged 6 weeks with a weight of 200 ± 10 g, were purchased from Shanghai Laboratory Animal Co., Ltd. (No. SCXK2023-0004; Shanghai, China). Rats were housed in an SPF room with a 12/12 h light/dark cycle and free access to food and water. The animals were acclimatized for 1 week before experiments. All animal experiments were approved by the Institutional Animal Care and Use Committee of Tongji Hospital of Tongji University (No. 2020-DW009).

### Study Design and Treatment Groups

**Fig. 1:**
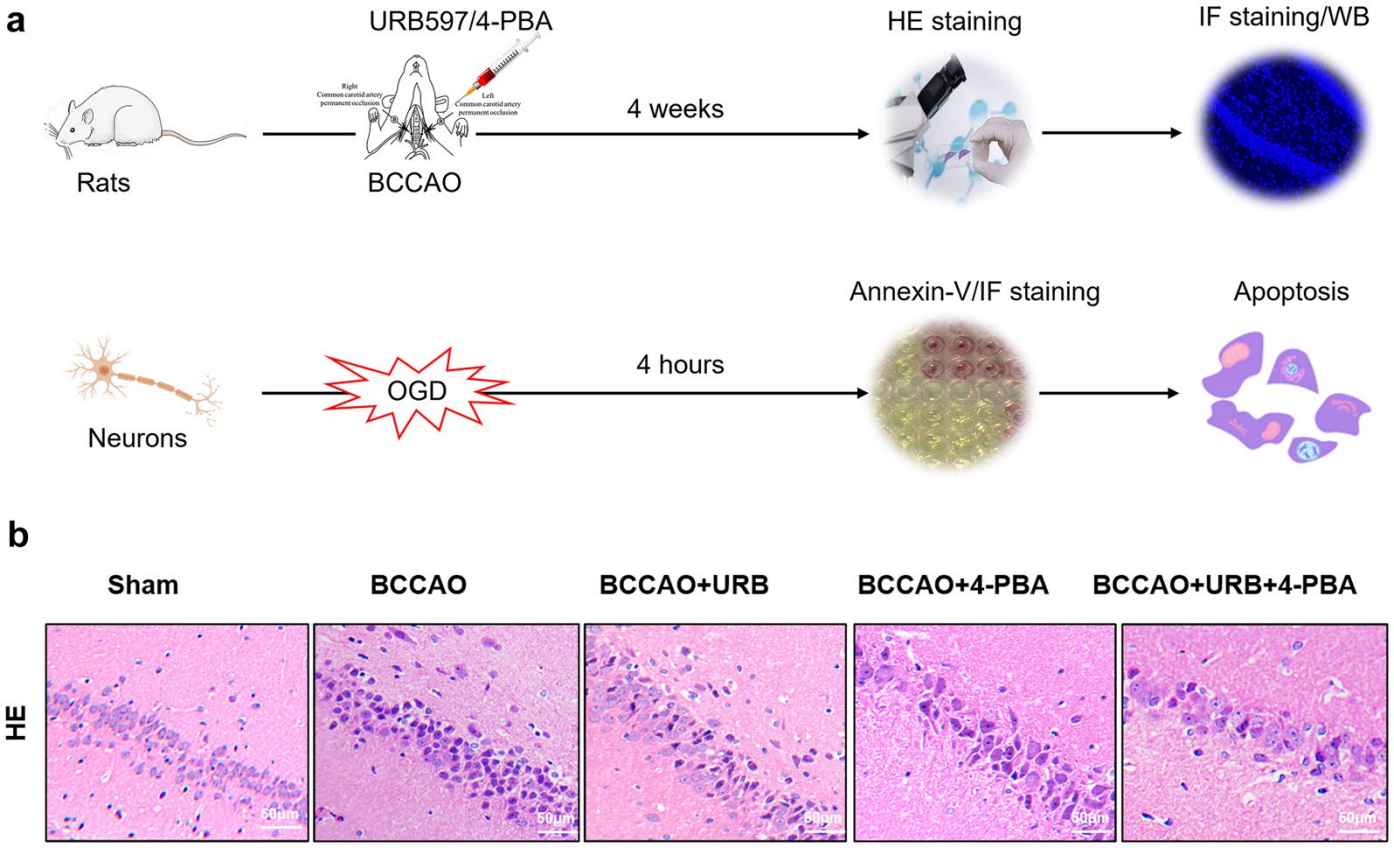
Effects of 4-PBA and URB597 on CCH-induced hippocampal neuronal morphological injury. **a** Experimental flow chart. CCH rats received drug interventions for 4 weeks and were then sacrificed for subsequent experiments. In the OGD experiments, HT22 cells were used. Cells received drug interventions for 0.5 h before OGD, underwent OGD for 4 h, and were then used for subsequent analyses. **b** Representative images of HE staining in the hippocampal CA1 area. (*n* = 4) scale bar: 50 μM.

The flow chart for the experimental procedure is shown in Fig. 1a. Firstly, rats underwent permanent bilateral common carotid artery occlusion (BCCAO) to induce CCH as previously described [11]. Briefly, rats were anesthetized using 3.5% chloral hydrate (400 mg/kg, intraperitoneal [i.p.]) and the bilateral common carotid arteries were exposed and occluded by double ligation with two 4–0 sutures in the median cervical incision. Rats in the sham group were subjected to the same surgical procedure without BCCAO. Five rats died due to anesthesia failure or surgical intolerance. CCH rats were treated with the ER stress inhibitor 4-PBA (No. HY-A0281, Med Chem Express, Shanghai, China) and the fatty‐acid amide hydrolase (FAAH) inhibitor URB597 (No. HY-10864, Med Chem Express, Shanghai, China). HT22 cells (mouse hippocampal neurons; ATCC, Manassas, VA, USA) were used in oxygen–glucose deprivation (OGD) *in vitro* experiments.

Rats were randomly assigned to the following five groups: (i) the sham group (Sham), (ii) the CCH model group (BCCAO), (iii) the 4-PBA treatment group (BCCAO+4-PBA), (iv) the URB597 treatment group (BCCAO+URB), and (v) the cotreatment group (BCCAO+URB+4-PBA) (*n* = 6 rats per group). Rats received daily injections of URB597 (0.3 mg/kg/day, i.p.) and 4-PBA (40 mg/kg/day, i.p.) for 4 weeks in the drug intervention groups. The dosages were selected based on previous studies [12–14]. Other rats received daily injections of an equal amount of vehicle. No rats died in the course of this intervention. Two hours after the last injection, the animals were euthanized for subsequent experiments.

Before OGD, HT22 cells were cultured in Dulbecco's modified Eagle's medium (Gibco, Carlsbad, USA) supplemented with 10% fetal bovine serum (Gibco, Carlsbad, USA) and 1% penicillin–streptomycin (Hyclone, UT, USA). Cells in the OGD model group were cultured in medium without sugar and serum and incubated at 37 °C in an incubator (Heraeus, Hanau, Germany) with a mixture of 95% N_2_ and 5% CO_2_ for 4 h to induce OGD. Then, the cells were cultured in complete medium at 37 °C with a mixture of 95% O_2_ and 5% CO_2_ for 24 h. Cells were randomly divided into the following five groups: (i) the control group (Con), (ii) the OGD model group (OGD), (iii) the 4-PBA intervention group (OGD+4-PBA), (iv) the GS-4997 (ASK1 inhibitor) intervention group (OGD+GS), and (v) the combination group (OGD+GS+4-PBA). The cells were preconditioned with 4-PBA (5 mM) and GS-4997 (10 μM) for 0.5 h before OGD [15].

### Hippocampal Hematoxylin and Eosin Staining

First, 4-μM-thick brain paraffin sections were prepared, and then hematoxylin and eosin (HE) staining was performed as previously described [3]. Briefly, samples were treated by routine deparaffinization and dehydration. Then slices were stained with hematoxylin for 5 min, followed by counterstaining with eosin for 10 s. Neurons in the hippocampal CA1 areas were photographed using a light microscope at 400× magnification (Olympus, Tokyo, Japan). Three cerebral slices of each animal were photographed to analyze their morphology by an investigator who was blinded to groups (n = 3).

### Immunofluorescence Staining

Paraffin-embedded brain sections were deparaffinized, immersed in EDTA-tris solution (pH 9.0) at 98 °C for 30 min, and washed three times with PBS for 5 min. In addition, HT22 cells were washed three times with PBS, permeabilized with 0.1% Triton X-100 (Sigma-Aldrich) in PBS for 5 min, and then blocked with 5% goat serum (Sigma-Aldrich) in PBS for 1 h. Subsequently, these sections and cells were incubated overnight at 4 °C with anti-GRP78 (No. ab212054, Abcam, 1:300) or anti-tubulin (No. 2146S, CST, 1:200). After washing, samples were incubated with Alexa Fluor 647-conjugated secondary antibody (1:200, Santa Cruz) at 37 °C for 1 h. After counterstaining with DAPI, samples were observed using fluorescence microscopy (Carl Zeiss LSM 700, Germany) by an investigator blinded to groups.

### Western Blot and Immunoprecipitation Analysis

The rat brains were lysed in RIPA buffer with protease inhibitor cocktail (Sigma). Protein concentrations were determined with the BCA Protein Assay Kit (No. P0012S; Beyotime, Shanghai, China). Proteins were separated by 6 − 12% SDS-PAGE (equal amounts per lane, 20–30 μg) and then transferred onto polyvinylidene difluoride membranes (Millipore, Billerica, MA). After blocking, the membranes were incubated with primary antibodies, including anti-GRP78 (No. ab212054, Abcam, 1:1000), anti-IRE1α (No. ab37073, Abcam, 1:1000), anti-TRAF2 (No. AF5327, Beyotime), anti-CHOP (No. DF6025, Affinity, 1:500), anti-Caspase-12 (No. sc-21747, Santa Cruz), anti-ASK1 (No. AF6477, Affinity, 1:1000), anti-p-ASK1 (No. AF3477, Affinity, 1:1000), anti-JNK1/2/3 (No. ab179461, Abcam, 1:1000), and anti-p-JNK1/2/3 (Abcam, 1:1000), at 4 °C overnight, washed, and incubated with suitable secondary antibodies (1:2000) at room temperature for 1 h. Protein bands were visualized using Western bright ECL solution (Millipore, Watford, UK) and analyzed using Image-Pro Plus 6.0 software (Bethesda, MD, USA).

For co-immunoprecipitation (Co-IP) analysis, the protein fraction was pretreated with a rabbit polyclonal anti-ASK1 antibody and a rabbit polyclonal anti-TRAF2 antibody. Protein A/G agarose (No. P2295M, Beyotime, Shanghai) was added to each sample and samples were incubated overnight at 4 °C. Human normal IgG (No. A7001, Beyotime, Shanghai). The mixture was rinsed with lysis buffer and then boiled, and the denatured immunocomplex solutions were analyzed by Western blot as previously described [16].

### TUNEL Assay

A CF488 TUNEL Cell Apoptosis Detection Kit (No. G1504, Servicebio, Shanghai) was used to assess the number of apoptotic cells according to the manufacturer’s instructions. Nuclei were stained for 5 min with 0.1 g/mL DAPI (No. G1012, Servicebio, Shanghai). TUNEL-positive cells were counted in three non-overlapping microscope fields in the hippocampal CA1 area at 200× magnification (Olympus, Tokyo, Japan).

### Flow Cytometry Analysis

The Annexin V-FITC apoptosis assay kit (No. C1062S, Beyotime, Shanghai) was used to detect apoptosis as follows. First, 1 × 10^6^ HT22 cells in the logarithmic phase growth were seeded in a six-well plate and given different treatments. The cells were washed three times with PBS, digested with trypsin, and collected by centrifugation. The cells were kept in the dark for 10 min and then propidium iodide was added. Finally, Annexin V-FITC was added, and the cells were kept in the dark for 5 min before analysis. Data were acquired and analyzed using Quest software (Becton–Dickinson). The data are expressed as a percentage of cells.

### RT-PCR

Total RNA was isolated from brain tissues using an RNA/DNA Isolation Kit (No. R0017S, Beyotime, Shanghai) and reverse-transcribed into cDNA with a cDNA Synthesis Kit (No. D7170S, Beyotime, Shanghai) according to the manufacturer’s instructions. RT primers used were synthesized by Sangon Biotech Company (Shanghai, China). Primer sequences were as follows: ***TNF-α***, forward: 5′-CGTCGTAGCAAACCACCAAGC-3′, reverse: 5′-CCAGTCGCCTCACAGAGCAAT-3′ (436 bp); ***IL-1β***, forward: 5′-ATAGCAGCTTTCGACAGTGAGG-3′, reverse: 5′-GGAGAATACCACTTGTTGGCTTA-3′ (447 bp); ***iNOS***, forward: 5′-ATCCCGAAACGCTACACTT-3′, reverse: 5′-TCTGGCGAAGAACAATCC-3′ (314 bp); ***GAPDH***, forward: 5′-GTTCAACGGCACAGTCAA-3′, reverse: 5′-CTCGCTCCTGGAAGATGG-3′ (77 bp). The PCR program was as follows: an initial denaturation step at 94 °C for 5 min, followed by 35 cycles of 1 min at 94 °C, 2 min at 60 °C, and 3 min at 72 °C. The mRNA expression levels were normalized to *GAPDH*.

### Statistical Analysis

The data were analyzed using the GraphPad Prism v.6.0 software (GraphPad Software Inc., USA) and the SPSS v.22.0 software (IBM, USA). Results are expressed as mean ± standard deviation. All data analyses were performed by one-way analysis of variance (ANOVA) followed by Tukey's post hoc test. Differences were considered significant when *P* < 0.05.

## Results

### 4-PBA and URB597 Ameliorate CCH-Induced Hippocampal Neuron Loss

The experimental flow chart is shown in Fig. 1a. Rats and HT22 cells were used for *in vivo* and *in vitro* experiments, respectively. As we previously reported, URB597 can protect neurons against stroke and improve hippocampal learning and memory. Here, as shown by HE staining of the hippocampal CA region, in the Sham group neurons were morphologically normal and tightly packed with abundant cytoplasm and clear nucleoli. The number of pyknotic nuclei and hyper-stained neurons was reduced in the URB597 treatment group compared to the BCCAO group (Fig. 1b). Similarly, cytoplasmic shrinkage and nucleus pyknosis were improved after treatment with 4-PBA and cotreatment with 4-PBA+URB597. These results suggest that 4-PBA and URB597 significantly improve CCH-induced hippocampal neuron loss, which is in line with previous studies emphasizing their neuroprotective effects.

### 4-PBA and URB597 Attenuate CCH-induced ER Stress

**Fig. 2:**
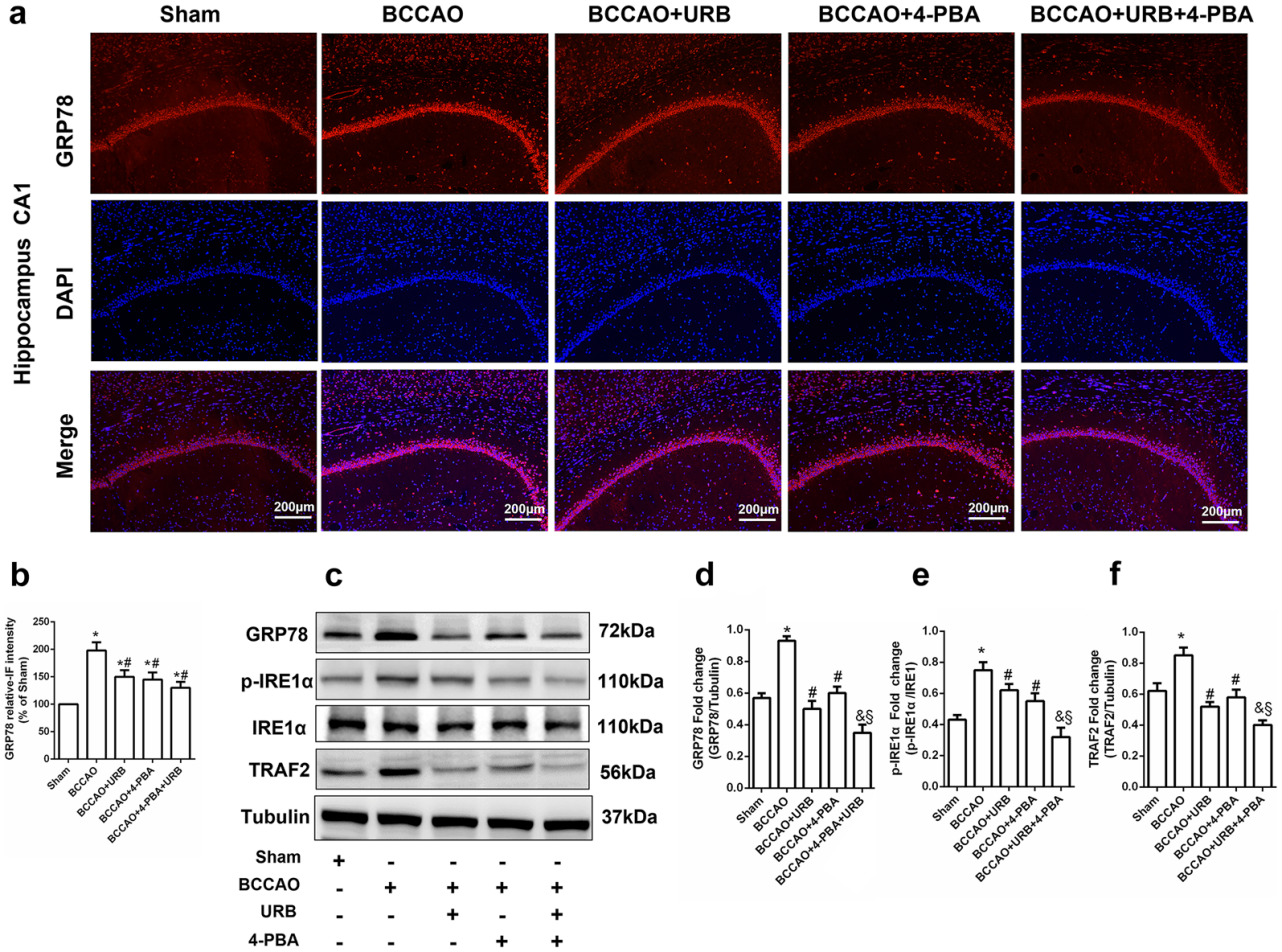
Effects of 4-PBA and URB597 on CCH-induced ER stress. **a** Representative images of GRP78 (red) immunofluorescence staining in the hippocampus. **b** Statistical analysis of the relative fluorescence intensity of GRP78. **c** The expression of ER stress signaling-related proteins as determined by Western blot, including GRP78, p-IRE1α, IRE1α, and TRAF2. **d**–**f** Protein expression levels. *P < 0.05 vs. Sham, ^#^*P* < 0.05 vs. BCCAO, ^&^*P* < 0.05 vs. BCCAO+URB, ^§^*P* < 0.05 vs. BCCAO+4-PBA (*n* = 3). scale bar: 200 μM.

Brain oxidative stress and the neuroinflammatory response are extremely easily caused by insufficient CBF and ischemic stroke [17]. The ER is a primary organelle responsible for reactive oxygen species (ROS) homeostasis associated with multiple cerebral pathological processes [18]. The effects of URB597 and 4-PBA on ER stress were further assessed using immunofluorescence staining and Western blot. The fluorescence signal of GRP78 in the hippocampus was enhanced in the BCCAO group compared to the Sham group and decreased by URB597, 4-PBA, and 4-PBA+URB597 (all *P* < 0.05, Fig. 2a, b). Western blot analysis yielded similar results for GRP78 (*P* < 0.05, Fig. 2c, d). IRE1α, p-IRE1α, and TRAF2 are biomarkers of ER stress. The phosphorylation levels of IRE1α were enhanced in the BCCAO group compared to the Sham group (*P* < 0.05, Fig. 2c, e). Western blot analysis revealed that URB597 and 4-PBA reduced TRAF2 expression (all *P* < 0.05, Fig. 2c, f). Taken together, these results suggest that CCH-induced excessive ER stress is attenuated by 4-PBA and URB597 treatment.

### 4-PBA and URB597 Suppress the CCH-Induced Inflammatory Response

**Fig. 3:**
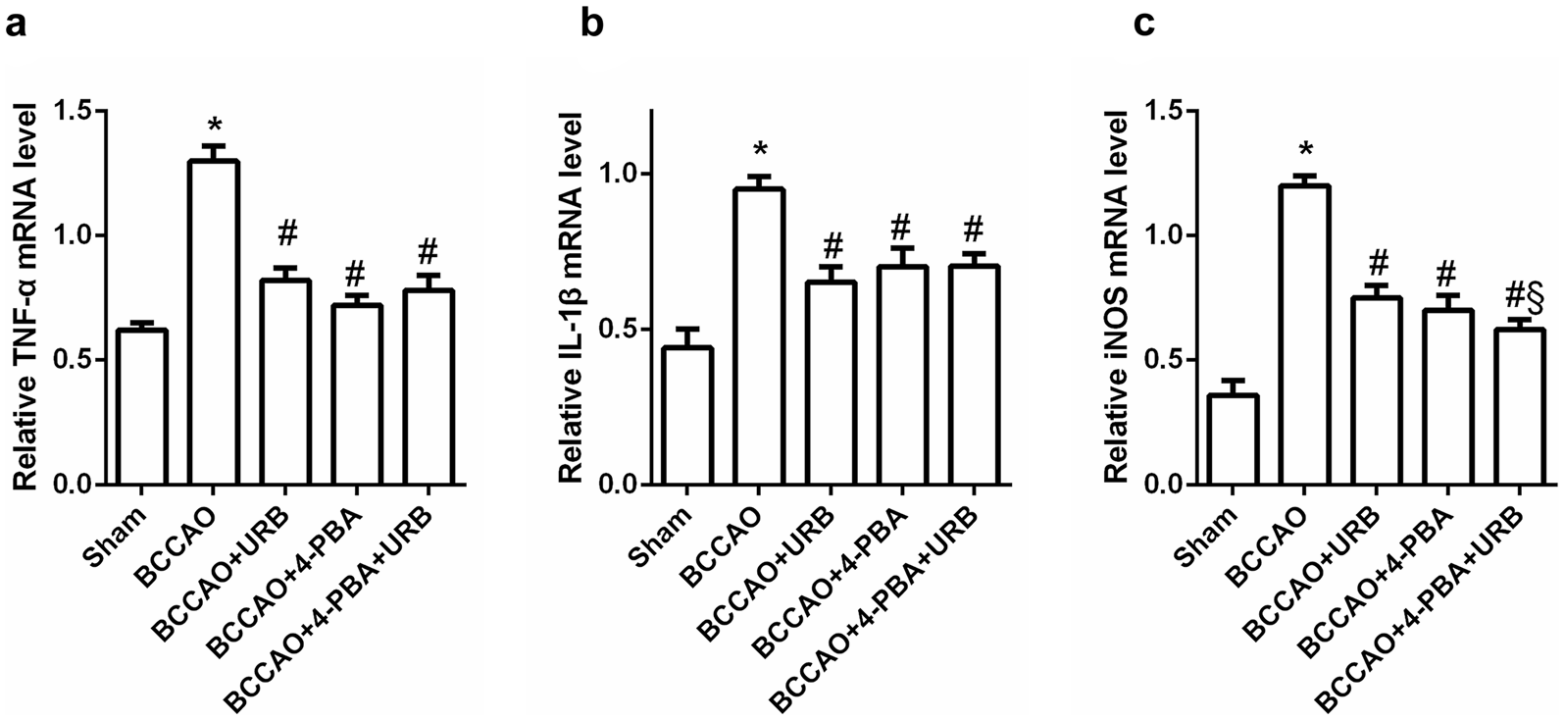
Effects of 4-PBA and URB597 on inflammatory cytokines. **a**–**c** Statistical analysis of the relative mRNA levels of the inflammatory cytokines TNF-α and IL-1β and iNOS. *P < 0.05 vs. Sham, ^#^*P* < 0.05 vs. BCCAO, ^&^*P* < 0.05 vs. BCCAO+URB, ^§^*P* < 0.05 vs. BCCAO+4-PBA (*n* = 4).

ER stress is an important factor in the inflammatory response that results in pathological processes [19]. Compared with the Sham group, the mRNA levels of the pro-inflammatory cytokines TNF-α and IL-1β and iNOS were increased in the BCCAO group (all *P* < 0.05, Fig. 3a). URB597, 4-PBA, and 4-PBA+URB597 reduced the mRNA levels of TNF-α, IL-1β, and iNOS (all *P* < 0.05, Fig. 3b, c). These data show that 4-PBA exerts anti-inflammatory effects by inhibiting ER stress and that URB597 can attenuate the CCH-induced inflammatory response.

### 4-PBA and URB597 Inhibit ER Stress-Mediated Hippocampal Neuronal Apoptosis in CCH

**Fig. 4:**
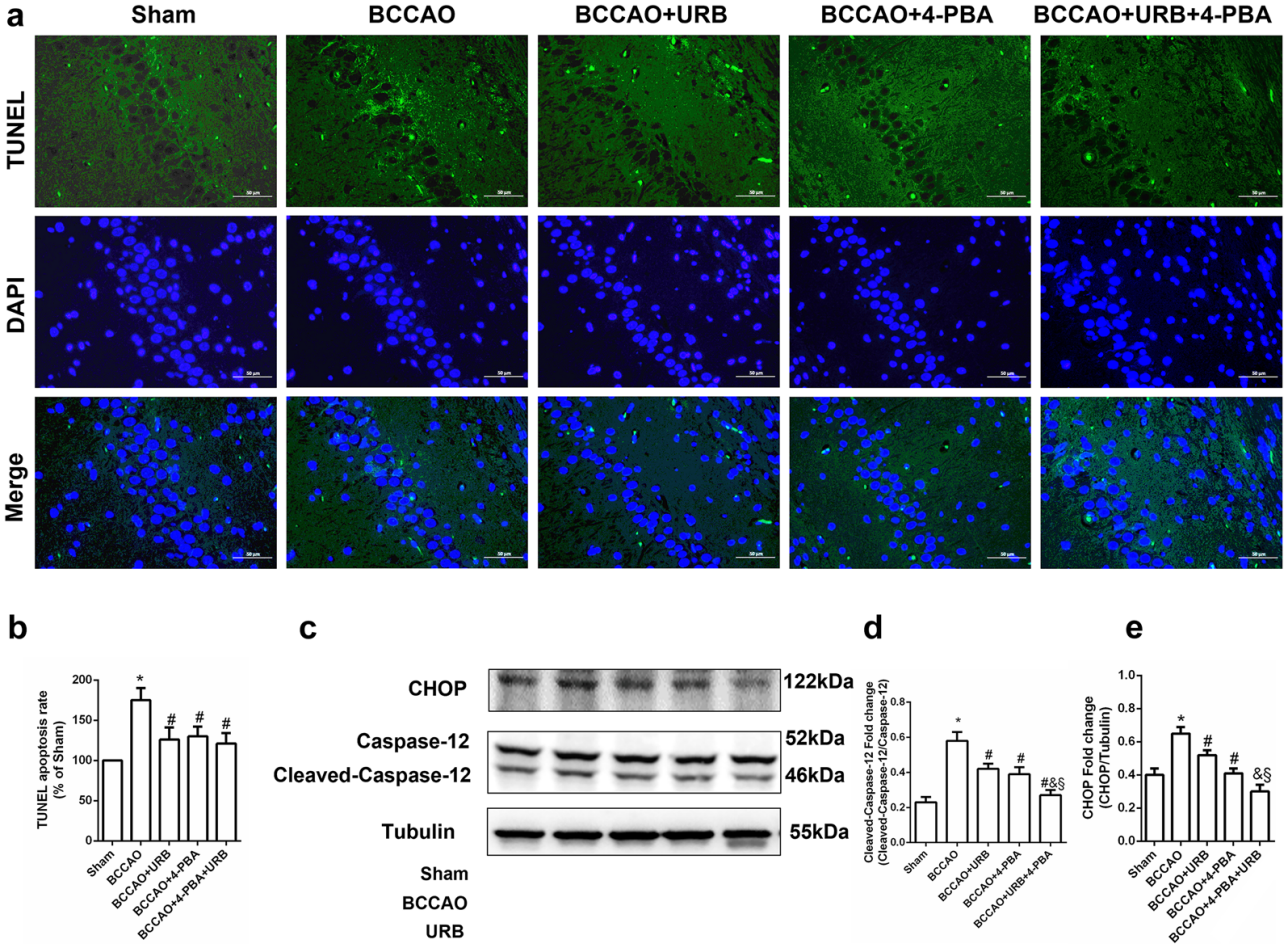
Effects of 4-PBA and URB597 on ER stress-related apoptosis. **a** Representative images of TUNEL (green) staining for neuronal apoptosis. **b** Statistical analysis of the proportion of TUNEL-positive cells. **c** Expression levels of the ER stress-related apoptosis markers Caspase-12 and CHOP. **d**, **e** Protein expression levels. *P < 0.05 vs. Sham, ^#^*P* < 0.05 vs. BCCAO, ^&^*P* < 0.05 vs. BCCAO+URB, ^§^*P* < 0.05 vs. BCCAO+4-PBA (*n* = 3). scale bar: 50 μM.

ER stress can be triggered by cerebral ischemia and hypoxia, leading to irreversible organelle damage and dysfunction, such as apoptosis [20]. To detect apoptotic cells, we performed a TUNEL labeling assay. Compared with the Sham group, the number of TUNEL-positive hippocampal neurons was increased in the BCCAO group (Fig. 4a, b). Caspase-12 plays a central role in the initiation of ER stress-induced apoptosis. Cleaved-Caspase-12 upregulation and processing have been observed after the ischemic episode (Fig. 4c). URB597 and 4-PBA reduced the number of TUNEL-positive cells and the expression level of cleaved-Caspase-12 (all *P* < 0.05, Fig. 4d). CHOP is another biomarker of ER stress-related apoptosis. Western blot analysis revealed similar inhibitory effects of URB597 and 4-PBA on CHOP expression (all *P* < 0.05, Fig. 4c, e). These data show that 4-PBA and URB597 can inhibit ER stress-induced hippocampal neuronal apoptosis in CCH.

### 4-PBA and URB597 Suppress CCH-Activated TRAF2/ASK1/JNK Signaling

**Fig. 5:**
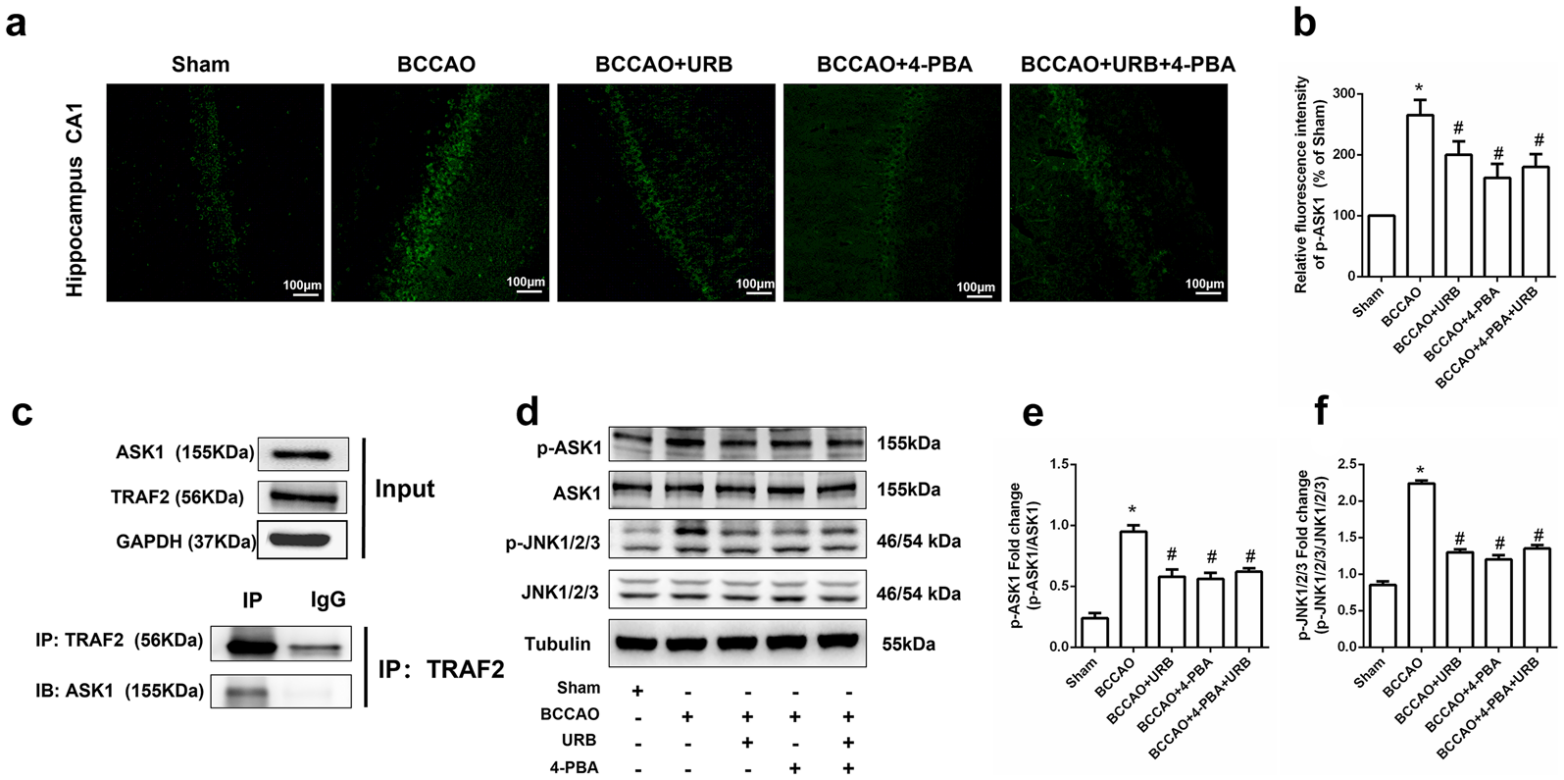
Effects of 4-PBA and URB597 on TRAF2/ASK/JNK signaling. **a** Representative images of p-ASK1 (green) immunofluorescence. **b** Statistical analysis of the relative fluorescence intensity. **c** Co-IP experiment between TRAF2 and ASK1. **d** Representative Western blots of the ASK/JNK signaling-related proteins ASK1, p-ASK1, JNK1/2/3, and p-JNK1/2/3. **e**, **f** Relative protein expression levels. *P < 0.05 vs. Sham, ^#^*P* < 0.05 vs. BCCAO, ^&^*P* < 0.05 vs. BCCAO+URB, ^§^*P* < 0.05 vs. BCCAO+4-PBA (*n* = 3). scale bar: 100 μM.

ASK1, also known as MAP3K5, is a serine/threonine kinase. It is a member of the MAP3K family that has been implicated in the pathology of neurodegenerative and oxidative stress-related diseases [21]. Immunofluorescence analysis showed that during CCH, p-ASK1 levels were increased (Fig. 5a). Our Co-IP experiment showed that ASK1 interacts with TRAF2 (Fig. 5c). Once activated, ASK1 acts as an upstream activator of JNK. Western blot analysis showed that the expression and activity of p-JNK were increased in the BCCAO group compared with the Sham group (Fig. 5d). Compared with the BCCAO group, the fluorescence intensity of p-ASK1 was significantly weakened in the URB597 and 4-PBA groups (all *P* < 0.05, Fig. 5a, b). Furthermore, Western blot data showed that compared with the BCCAO group, the levels of p-ASK1 and p-JNK, which are involved in the ASK1/JNK signal transduction cascade, were decreased in the URB597, 4-PBA, and 4-PBA+URB597 groups (all *P* < 0.05, Fig. 5e, f). These data revealed that CCH enhanced TRAF2/ASK1/JNK signaling and that 4-PBA and URB597 may inhibit this pathway activation.

### 4-PBA and GS-4997 Exert Synergistic Anti-Apoptotic Effects on HT22 Cells with OGD

**Fig. 6:**
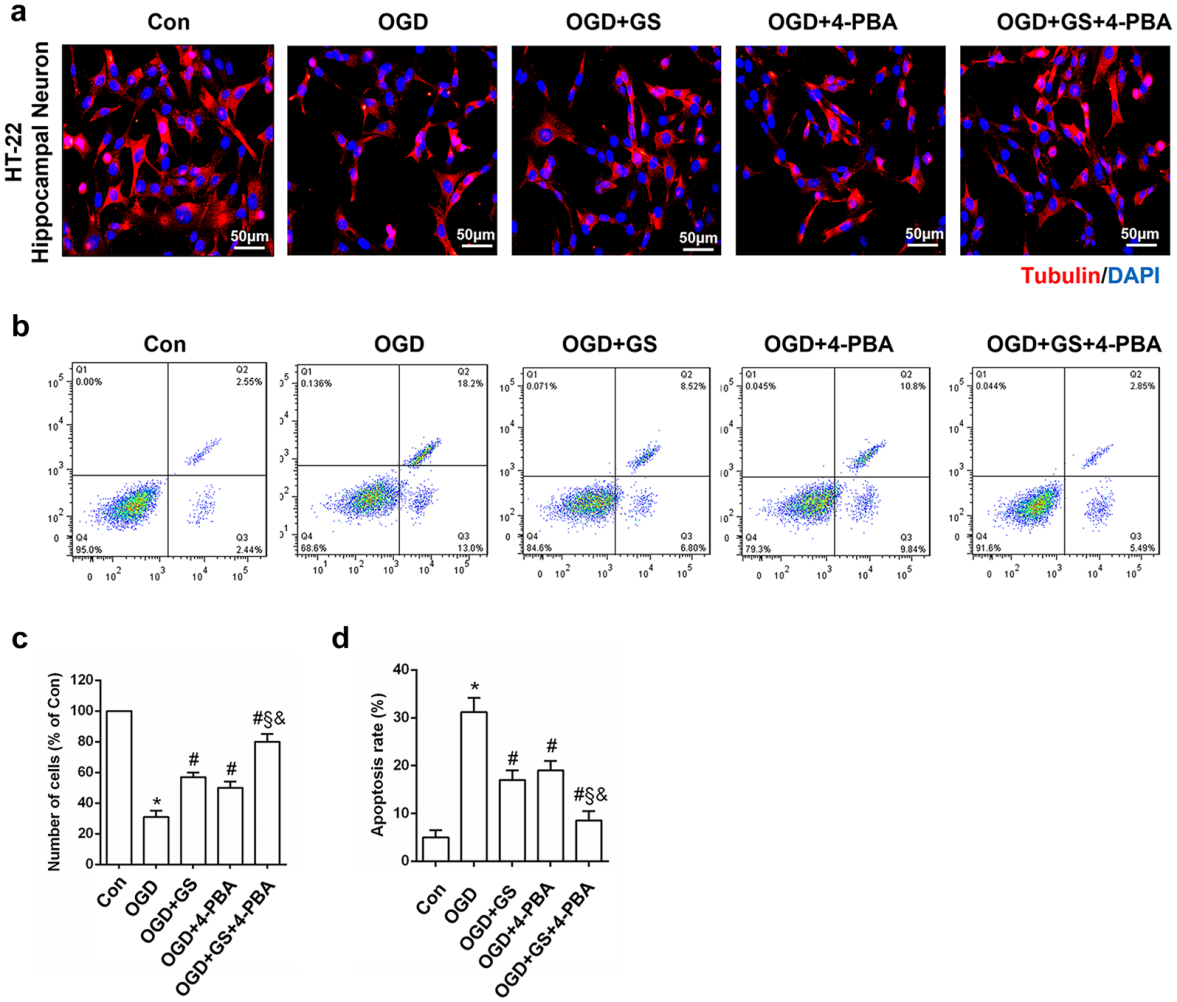
Effects of 4-PBA and GS-4997 on HT22 cell survival with OGD. **a** Representative images of tubulin immunofluorescence (red) and DAPI staining (blue). **b** Cellular apoptosis was detected by Annexin V-FITC/propidium iodide flow cytometry analysis. **c**, **d** Protein expression levels. *P < 0.05 vs. Con, ^#^*P* < 0.05 vs. OGD, ^&^*P* < 0.05 vs. OGD+4-PBA, ^§^*P* < 0.05 vs. OGD+GS (*n* = 4). Magnification: 200×; scale bar: 50 μM.

ASK1 can activate a cascade of pathological events in brain ischemia [22, 23]. To further clarify the role of ER stress as well as the role of ASK1 in cellular injury, we studied the effects of 4-PBA and GS4997 on HT22 cell survival (mouse hippocampal neurons) using an *in vitro* model of stroke (OGD) (Fig. 6a). In the OGD group, the proportion of apoptotic cells was greatly increased compared with the control group (*P* < 0.05, Fig. 6b, c). Following pretreatment with 4-PBA or GS4997, the proportion of apoptotic cells was reduced to 15.3 ± 5.2% and 21.6 ± 4.5%, respectively. The anti-apoptotic effect of cotreatment was more effective than that of single treatment (*P* < 0.05, Fig. 6d). These findings suggest that ASK1 is involved in ischemic ER stress-induced neuronal damage and that 4-PBA and GS-4997 could mitigate OGD-induced hippocampal neuronal apoptosis.

## Discussion

BCCAO decreases CBF by 35%–45% in the cortex and by 60% in the hippocampus [24]. How do neurons respond to this and what pathological changes may occur in them? Neurons initiate a series of highly conserved self-protection mechanisms to cope with the stress, such as autophagy, mitochondrial quality control, and the UPR. ER-associated protein degradation is known to be a key mechanism integral to ER protein quality control triggered by the UPR [25]. However, if misfolded proteins are not sufficiently degraded, neurons activate ER stress, possibly followed by ER stress-induced apoptosis. ER stress has been implicated in neuronal protein degeneration in AD [26]. Our data demonstrated that inhibition of ER stress by 4-PBA and URB597 reduces the generation of inflammatory cytokines and neuronal cell death in the hippocampus. It has been reported that ER stress and the UPR in inflammatory processes result in synapse failure and neuronal injury in vascular dementia [27]. In CCH pathological processes, inflammatory cascades exacerbate ER damage, causing increased ROS production, thus initiating a vicious cycle [1]. There is a complex crosstalk among inflammation, oxidative stress, and ER stress, which can initiate and aggravate chronic diseases [19, 28].

In the present study, we first confirmed that URB597 and 4-PBA inhibit CCH-induced ER stress. URB597 (KDS-4103, cyclohexyl carbamic acid 3′-carbamoyl-3-ylester), a highly selective inhibitor of the enzyme FAAH, shows significant anti-inflammatory effects with an IC_50_ of approximately 0.5 nM in rat neurons [29–31]. 4-PBA, a specific inhibitor of ER stress, is usually used in infection and cancer studies. These inhibitors exert biological effects primarily by regulating endogenous signal transduction. First, URB597 was reported to have therapeutic potential for treating posttraumatic stress disorder, inflammatory pain, alcoholic cognitive impairment, and oxidative stress-related diseases [32–35]. Recently, it was found that in transient brain ischemia, URB597 exerts neuroprotective effects on mouse neurons in a time-dependent and dose-dependent manner by regulating autophagic flux and necroptosis [36]. Second, 4-PBA decreased infarct volume, alleviated blood–brain barrier disruption, and protected neurons against apoptosis in a rat stroke model [37]. 4-PBA also attenuates primary neuron death during OGD [38]. We previously reported that URB597 can protect primary cultured hippocampal neurons against OGD, and CCH-induced neuroinflammation and autophagy dysfunction were attenuated by URB597 [13, 14]. A growing body of evidence indicates that URB597 and 4-PBA have antioxidant and anti-inflammatory effects. In spinal cord injury, 4-PBA was demonstrated to inhibit necroptosis of microglia/macrophages [39]. URB597 improves hippocampal neuron survival by reducing oxidative stress and inflammation in a rat model of AD [40]. Here, we found that CCH-induced hippocampal neuronal loss and apoptosis were attenuated by URB597, 4-PBA, and 4-PBA+URB597, as shown by HE staining and TUNEL staining assays (Figs. 1 and 2). In addition, the hallmarks of ER stress-related apoptosis in CCH, CHOP and Caspase-12, were partly suppressed by URB597 and 4-PBA, suggesting that they exert a therapeutic effect against ER stress in chronic cerebral ischemia.

The MAPK and PI3K/Akt signaling pathways are interconnected and irreplaceable for cell survival, governing cellular activities in response to stress. The MAPK cascade participates in a myriad of signaling pathways, regulating signal transduction from input to output in brain ischemia [41, 42]. ASK1, a serine/threonine kinase that is a member of the MAP3K family, is involved in the regulation of cell survival, proliferation, inflammation, and apoptosis via activating JNK and p38 [43]. Our previous research has shown that cannabinoid-profiled agents or cannabinoid receptor agonists (WIN55, 212–2) ameliorate CCH-induced mitochondrial damage in hippocampal neurons by inhibiting the p38 pathway [23]. The MAPK signaling pathway regulates CCH pathological processes by enhancing ASK1 and JNK phosphorylation. The ASK1/JNK signaling pathway may be an instigator of detrimental injury after CCH. ASK1 cannot be activated by only ROS or oxidative stress but also depends on regulation by TNF-α [44, 45]. In addition, ASK1 and TRAF2 can be recruited by activated IRE1α on the ER membrane in prolonged ER stress and thus activate the ASK1-dependent apoptosis pathway [46]. In the present study, the protein levels of IRE1α and TRAF2 were increased in the BCCAO group, which suggests that the ER stress signaling cascade was activated by brain ischemia. Co-IP experiments showed that ASK1 was activated by TRAF2 through formation of an IRE1α–TRAF2–ASK1 complex. Early studies showed that this complex can induce apoptosis in cancer cells [16]. In *in vitro* OGD experiments, the ER stress inhibitor 4-PBA and the ASK1 inhibitor GS-4997 could simultaneously reduce neuronal death. These results indicate that ASK1 is involved in ER stress-induced apoptosis in CCH. *ASK1* conventional knockout mice do not exhibit developmental and cognitive defects [47], and blockade of this cascade, by either pharmacological or genetic manipulation, decreases neuronal cell death in neurodegenerative diseases and ischemic cerebrovascular diseases [48–50], which is in line with our findings. Therefore, targeting ASK1 in ER stress-induced apoptosis may mitigate ischemic neuronal injury.

Some limitations should be pointed out. First, changes in pro-inflammatory cytokines were only assessed at the mRNA level. Their protein expression levels should also be investigated. Second, the ER is one of the most important organelles for ROS production [18]. It would be interesting to analyze intracellular oxidative stress by testing the levels of ROS, catalase, and malondialdehyde. In addition, our demonstration of hippocampal neuron preservation would benefit from cognitive behavioral tests such as the Morris water maze test or a novel object recognition test, because the hippocampus is the most important brain area for forming and maintaining learning and memory [51]. These limitations should be addressed in future animal experiments.

## Conclusion

**Fig. 7:**
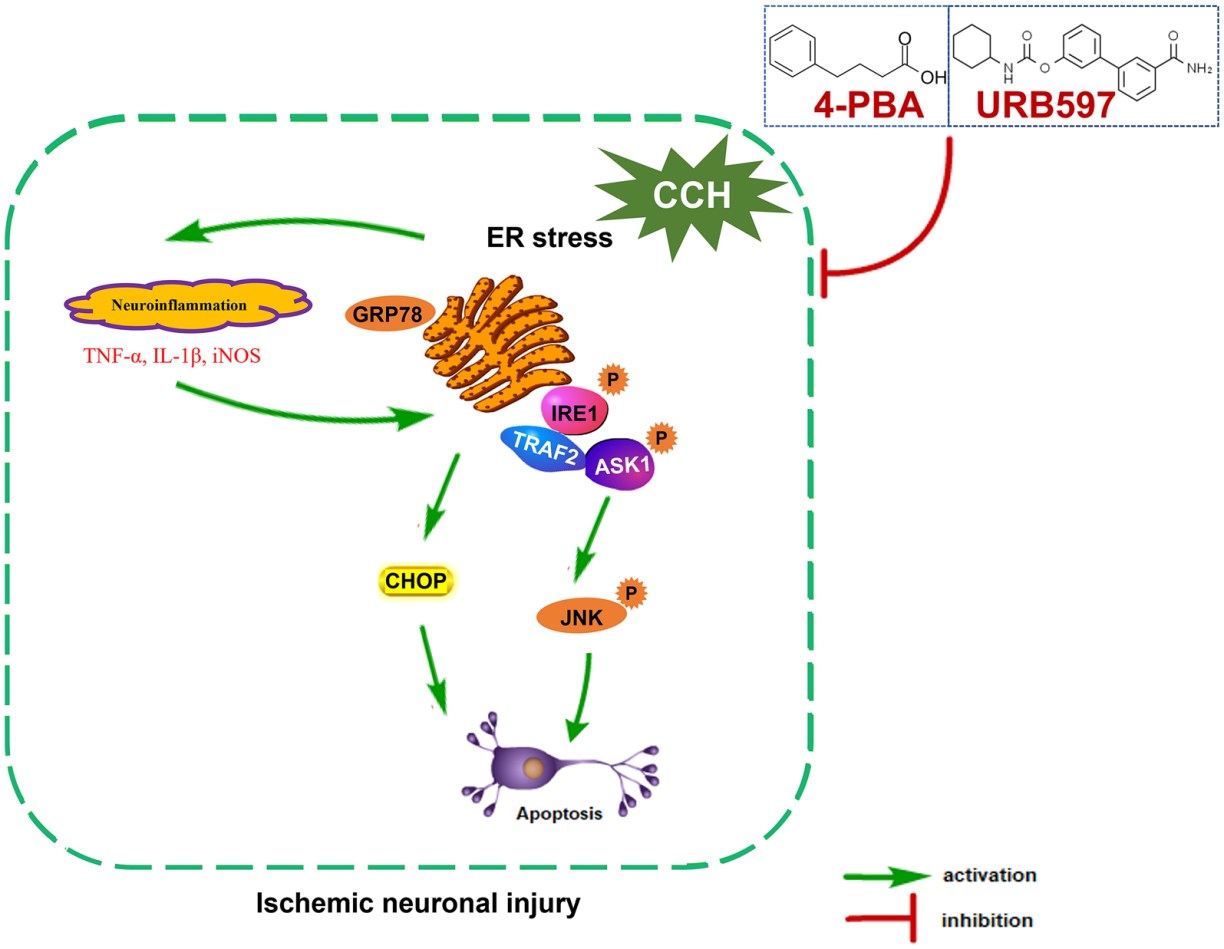
Schematic showing how inhibition of ER stress exerts neuroprotective effects on cerebral ischemic neuronal injury.

Our current study revealed for the first time that inhibition of ER stress exerts significant neuroprotective effects through alleviating IRE1α/TRAF2/ASK1/JNK signaling in CCH-induced hippocampal neuronal injury (Fig. 7). Targeting the ER stress–ASK1 cascade is a novel therapeutic approach for treating ischemic cerebrovascular diseases.

## Data Availability

Data will be made available upon reasonable request.
